# Troponin T1 in tumorigenesis and immune modulation: Insights into multiple cancers and kidney renal clear cell carcinoma

**DOI:** 10.1111/jcmm.18410

**Published:** 2024-06-09

**Authors:** Qianjin Zhang, Lin Hao, Fengye Wang, Quansheng Yu, Shaoyuan Wu, Conghui Han

**Affiliations:** ^1^ Department of Urology, Xuzhou Central Hospital Affiliated Central Hospital of Xuzhou Medical University Xuzhou Jiangsu China; ^2^ Department of Urology The Affiliated Suqian First People's Hospital of Nanjing Medical University Suqian Jiangsu China; ^3^ School of Life Sciences Jiangsu Normal University Xuzhou Jiangsu China

**Keywords:** KIRC, prognosis, TNNT1, tumorigenesis, tumour microenvironment

## Abstract

Troponin T1 (TNNT1) plays a crucial role in muscle contraction but its role in cancer, particularly in kidney renal clear cell carcinoma (KIRC), is not well‐understood. This study explores the expression, clinical significance and biological functions of TNNT1 in various cancers, with an emphasis on its involvement in KIRC. We analysed TNNT1 expression in cancers using databases like TCGA and GTEx, assessing its prognostic value, mutation patterns, methylation status and functional implications. The study also examined TNNT1's effect on the tumour microenvironment and drug sensitivity in KIRC, complemented by in vitro TNNT1 knockdown experiments in KIRC cells. TNNT1 is overexpressed in several cancers and linked to adverse outcomes, showing frequent upregulation mutations and abnormal methylation. Functionally, TNNT1 connects to muscle and cancer pathways, affects immune infiltration and drug responses, and its overexpression in KIRC is associated with advanced disease and reduced survival. Knocking down TNNT1 curbed KIRC cell growth. TNNT1's aberrant expression plays a significant role in tumorigenesis and immune modulation, highlighting its value as a prognostic biomarker and a potential therapeutic target in KIRC and other cancers. Further studies are essential to understand TNNT1's oncogenic mechanisms in KIRC.

## 1 INTRODUCTION

Troponin T1, also known as TNNT1, is primarily responsible for encoding the troponin complex.^1^ It plays a crucial role in the precise regulation of muscle contraction by orchestrating the encoding of slow muscle‐type troponin T in physiological contexts.^2^, ^3^ Through intricate interactions with other subunits within the troponin complex, TNNT1 is involved in the intricate control of both muscle contraction and relaxation.^4^, ^5^ Abnormalities in the TNNT1 gene can disrupt the function of the troponin complex, leading to disturbances in muscle contraction coordination. These disruptions can manifest as symptoms such as muscle weakness, fatigue and restricted mobility.^6^, ^7^ However, the extent of TNNT1's role beyond conventional muscle tissue remains largely unknown.

Recent research has highlighted unusual TNNT1 expression in various cancerous growths, suggesting a potential involvement of TNNT1 in tumour development.^8^, ^9^, ^10^, ^11^, ^12^ For instance, in a comprehensive investigation of breast cancer, researchers observed a significant increase in TNNT1 levels in breast cancer cells, indicating a strong association with clinical stage, T, and N categories. Experimental studies further revealed that TNNT1 played a significant role in promoting the growth of breast cancer cells, particularly in facilitating the transition from G1 to S phase. Similarly, in a study on colorectal cancer, TNNT1 was found to be more prevalent in cancerous tissue, and its presence was linked to a poor prognosis for patients. Functional analyses suggested that TNNT1 might contribute to the advancement of colorectal cancer by facilitating the epithelial‐mesenchymal transition (EMT) process. However, further clarification is needed to fully understand the molecular mechanisms underlying TNNT1 dysregulation in different types of cancer and its subsequent effects on tumour biology.

Cancer constitutes a significant source of both illness and death on a global scale, with a projected 2 million new cases and 610,000 fatalities anticipated in 2024.^13^ Despite advances in treatment modalities, mortality rates remain high, primarily owing to late diagnosis and disease recurrence attributed to treatment resistance.^14^, ^15^, ^16^, ^17^ An imminent imperative exists to unearth innovative biological insights and prognostic/predictive markers that can adeptly facilitate precision‐based management for individuals grappling with cancer.^18^, ^19^ The intracellular signalling pathways governing cancer initiation and progression are highly complex, involving dysregulated expression of various structural and regulatory proteins.^20^, ^21^, ^22^ Elucidating the participation of unexpected proteins, such as TNNT1, in oncogenesis could foster a deeper understanding of tumour biology and facilitate the discovery of novel therapeutic targets. Tumour immunotherapy has revolutionized cancer treatment by leveraging the immune system's ability to combat malignancies. Immune checkpoint inhibitors, such as anti‐CTLA‐4 and anti‐PD‐1 antibodies, have demonstrated remarkable clinical success by blocking inhibitory pathways that suppress anti‐tumour immune responses.^23^ Additionally, chimeric antigen receptor (CAR) T‐cell therapy involves genetically engineering patients' T cells to express synthetic receptors targeting tumour‐associated antigens, enabling potent cancer cell elimination.^24^, ^25^ Identifying novel targets related to tumour immunology is one of the key research focuses.

In this study, we employed various databases, tools and techniques to perform an in‐depth analysis of TNNT1, with a specific emphasis on its expression, clinical relevance, role, genetic changes, immune cell presence, drug response and single‐cell investigations in human malignancies, specifically in kidney renal clear cell carcinoma (KIRC). Our comprehensive multi‐omics analysis yielded unprecedented insights into the tumour‐promoting activities of TNNT1 across various cancer types. Notably, in KIRC, TNNT1 emerged as a potential driver of malignant progression, immunotherapy response, and metabolic reprogramming. In addition, controlling the levels of TNNT1 was discovered to impact the growth and response to drugs of KIRC cell cultures in a laboratory setting. Collectively, these findings underscore the clinical importance of TNNT1 as an emerging prognostic biomarker and a potential target for therapeutic intervention, warranting meticulous exploration in the realm of targeted cancer prevention and treatment strategies. The flowchart of the study is shown in Figure S1.

## 2 MATERIALS AND METHODS

### 2.1 Gene expression analysis

Data on TNNT1 mRNA expression in healthy human tissues were obtained from the Genotype‐Tissue Expression Project (GTEx) database.^26^ Data on gene expression, genetic mutations and clinical information for cancer patients and healthy individuals were obtained from the UCSC Xena database, which includes 10,327 samples from cancer patients and 730 samples from individuals without any health issues.^27^ We used the R software tool ‘UCSCXenaShiny’ to analyse and visualize the normalized gene expression data for TNNT1 in cancerous and normal tissues from the UCSC Xena databases.^28^

### 2.2 Survival analysis

We examined the associations between TNNT1 mRNA expression levels and various survival metrics, including overall survival (OS), disease‐specific survival (DSS), disease‐free interval (DFI), and progression‐free interval (PFI), across different cancer types in the UCSC database. The number of samples analysed for each cancer type ranged from 57 (DLBC) to 1093 (BRCA). Survival analyses were performed using Kaplan–Meier and Cox regression methods.

### 2.3 Genetic alteration analysis

To explore the TNNT1 variant profile across multiple cancer types, we utilized the cBioPortal website.^29^ Using the ‘cancer types summary’ module, we gathered information on the occurrence of changes in frequency, types of mutations, and copy number alterations (CNAs) in every TCGA tumour. The types of mutations observed include missense mutations, nonsense mutations, frameshift mutations, and mRNA high. These mutation types are explained as follows: Missense mutations: These mutations result in a single amino acid change in the protein sequence encoded by the TNNT1 gene. Nonsense mutations: These mutations introduce a premature stop codon, leading to the production of a truncated and usually non‐functional protein. Frameshift mutations: These mutations cause a disruption in the reading frame of the gene, resulting in the alteration of the amino acid sequence downstream of the mutation site. mRNA high: this refers to an increase in the expression level of TNNT1 mRNA in tumour samples compared to normal samples.

### 2.4 Analysis of tumour mutational burden and microsatellite instability

An analysis was conducted to assess the correlation between TNNT1 expression and MSI and TMB, considering the increasing evidence linking cancer prognosis with these factors.^30^, ^31^ Microsatellite instability (MSI) and tumour mutational burden (TMB) scores were calculated using the TCGA pan‐cancer mutation dataset available at https://tcga.xenahubs.net. The correlation between TNNT1 expression levels and MSI/TMB was explained using Pearson's correlation coefficient. The conclusive outcomes were visually presented through radar plots generated using the R package ‘fmsb’.

### 2.5 Methylation analysis

Deviant gene methylation patterns may lead to the uncontrolled activation of genes linked to cancer.^32^ Methylation, which involves the addition or removal of methyl groups from DNA, can affect gene expression by regulating the accessibility of DNA to transcription factors and other regulatory proteins. Methylation data for tumour and normal samples were acquired from the UCSC Xena dataset. The differential statistics and visualization of TNNT1 methylation level differences in cancerous and non‐cancerous tissues were performed using the R package ‘UCSCXenaShiny’.

### 2.6 Functional enrichment analysis

Obtaining the top 50 relevant genes linked to TNNT1 was done by utilizing the similar genes feature in the GEPIA2 database at http://gepia2.cancer‐pku.cn/.^33^ A network of protein interactions was created using the STRING database at https://string‐db.org.^34^ Furthermore, gene–gene interaction networks were created utilizing the GeneMANIA database available at http://www.genemania.org.^35^ The Venn diagram created on the website (https://bioinformatics.psb.ugent.be/webtools/Venn/) displayed the overlap between the three datasets and highlighted the most important molecules. Using the R package ‘clusterProfiler’, subsequent analyses were performed on the combined genes from the mentioned datasets to elucidate the possible roles of TNNT1 through GO and KEGG methods. The Cancer Single‐Cell State Atlas (CancerSEA) database was used to thoroughly assess the relationship between TNNT1 expression and the functional statuses of various cancer cells.^36^

### 2.7 Immune cell infiltration analysis

The infiltration of immune cells was analysed using the TIMER, EPIC and MCPcounter algorithms. The Pearson correlation technique was used to examine the relationship between TNNT1 expression and immune cells.

### 2.8 Drug sensitivity analysis

The relationship between TNNT1 gene expression and drug sensitivity was evaluated using the CellMiner platform at https://discover.nci.nih.gov/cellminer/.^37^ Correlations were computed using the Pearson correlation method, implemented through the ‘Corrplot’ R package.

### 2.9 Clinical characteristics analyses of TNNT1 in KIRC

In KIRC patients from the TCGA database, the relationship between TNNT1 expression and various clinical characteristics such as gender, age, TNM stage, clinical stage and pathological grade was investigated. Numerical variables were analysed using the Mann–Whitney *U‐*test, and categorical variables were examined with either chi‐square or Fisher's exact tests.

### 2.10 Independent prognostic analysis and nomogram construction

Cox regression analysis was utilized to discern independent prognostic factors. Afterwards, all independent prognostic factors that were identified were combined to create a nomogram, and calibration curves were produced to analyse and evaluate the nomogram's effectiveness, using the ‘RMS’ R package.

### 2.11 Enrichment analysis of TNNT1 in KIRC

Patients with KIRC were divided into groups based on high and low expression of TNNT1, determined by the median level of expression. DEGs were identified between these groups using the R package ‘limma’ and then analysed with GO and KEGG through the R package ‘clusterprofiler’. Additionally, the process of gene set enrichment analysis (GSEA) was carried out and displayed using the R tool ‘fgsea’. Results with an enrichment *p*‐value/adjusted *p*‐value less than 0.05 were considered to be statistically significant.

### 2.12 Immune landscape of TNNT1 in KIRC

The tumour microenvironment (TME) plays a crucial part in the development and treatment of KIRC.^38^ Various algorithms, including ESTIMATE, CIBERSORT and ssGSEA methodologies, were used to assess the relationship between TNNT1 expression and immune factors in the TME.

### 2.13 Response to targeted therapy and immunotherapy

Targeted therapies and immunotherapies are crucial modalities for managing KIRC.^39^ First, drug sensitivity to several commonly used targeted therapies was calculated using the pRRophetic algorithm. Furthermore, we performed investigations to examine the relationship between TNNT1 levels of expression and responsiveness to specific treatment, as indicated by IC50 measurements. We evaluated how patients with KIRC responded to immune checkpoint inhibitors (ICIs) by analysing immunophenoscore (IPS) data from The Cancer Immunome Atlas (TCIA) database and TMB data from TCGA database. Afterwards, we analysed the differences in IPS and TMB values among the groups with elevated and reduced TNNT1 expression levels. Generally, elevated IPS and TMB values signify a more favourable response to immunotherapy.

### 2.14 Single‐cell analysis of TNNT1 in KIRC

Single‐cell dataset of KIRC (GSE159115) was analysed based on the TISCH database (http://tisch.comp‐genomics.org/)^40^ to visualize TNNT1 expression and distribution in KIRC.

### 2.15 Cultured cell lines

The KIRC cell lines ACHN, 786‐O, and Caki‐1, as well as the normal renal cell line HK‐2 (ATCC), were cultured at 37°C with 5% CO_2_.ACHN and 786‐O were grown in RPMI 1640 medium from Gibco, whereas Caki‐1 was cultivated in McCoy's 5A medium from Gibco. The maintenance of HK‐2 cells was conducted in DMEM/F12 medium (Gibco). All culture media were enriched with 10% fetal bovine serum (FBS) (Hyclone) and 1% penicillin–streptomycin.

### 2.16 Extraction of RNA and measurement using quantitative real‐time polymerase chain reaction

KIRC cell lines were used to extract total RNA with the RNA‐easy isolation reagent from Thermo Fisher Scientific, followed by cDNA synthesis using the PrimeScript RT Master Mix from Takara Bio, Inc. according to the provided instructions. Quantitative real‐time polymerase chain reaction (qRT‐PCR) was used to analyse gene expression, with the Taq Pro Universal SYBR qPCR Master Mix (Vazyme, Q712‐02) employed. To ensure accurate normalization, GAPDH was used as a reference control, and the levels of the target gene expression were calculated through the 2‐ΔΔCt technique. The primer sequences utilized for GAPDH and TNNT1 are provided in the Additional file: Table S1.

### 2.17 Introduction of small interfering RNA into cells

Small interfering RNAs (siRNAs) designed to target TNNT1 and non‐targeting negative controls (NC) were obtained from Gene Pharma and delivered into KIRC cells with Lipofectamine 3000 according to the manufacturer's instructions. The target sequences for TNNT1 siRNA are detailed in the Additional file: Table S2.

### 2.18 Statistical analysis

Statistical analyses were executed employing R software (version 4.3.1).Group differences were assessed using either the Student's *t*‐test or the Mann–Whitney *U*‐test for continuous variables, and categorical variables were analysed with the chi‐square or Fisher's exact tests. Correlations were evaluated using Pearson's correlation coefficient. Statistical criteria deemed *p*‐values below 0.05 to be significant.

## 3 RESULTS

### 3.1 Expression analysis of TNNT1 across diverse types of cancer

**FIGURE 1:**
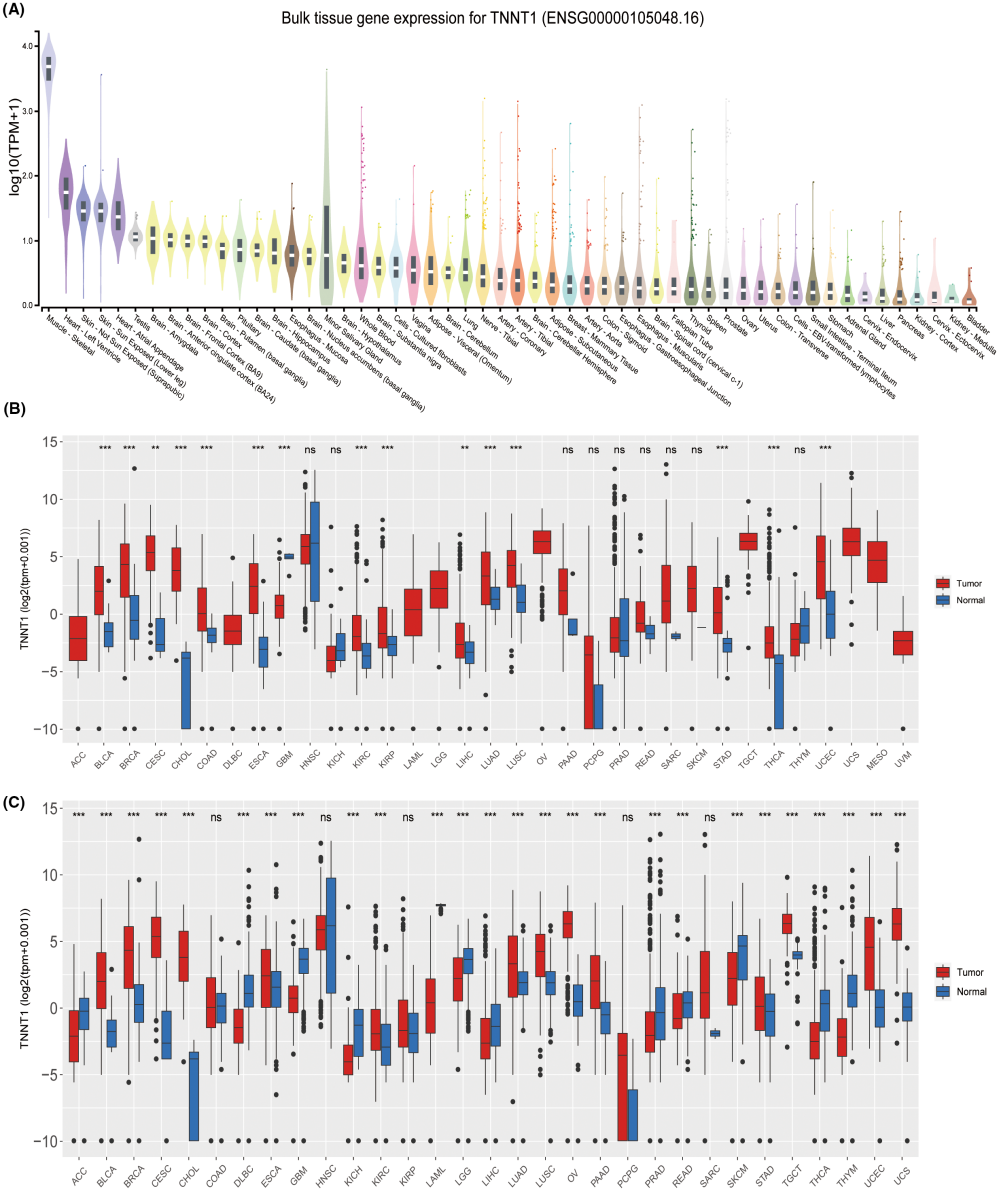
Pan‐cancer TNNT1 expression. (A) Bulk tissue gene expression for TNNT1; (B) pan‐cancer expression of TNNT1 in the TCGA database; (C) pan‐cancer expression of TNNT1 in the TCGA and GTEx databases. ns, not significant; **p < 0.01; ***p < 0.001.

Distinct patterns of TNNT1 expression were observed in different tissues during the examination. Higher levels of expression were observed in tissues like skeletal muscle, heart and skin, with lower levels detected in organs like the bladder, kidney and cervix (Figure 1A). Moreover, an examination of TCGA data across multiple types of cancer revealed a notable increase in TNNT1 expression in 15 different tumours (Figure 1B). Our analysis consistently found significant upregulation of TNNT1 in 14 different types of tumours by integrating data from TCGA and the GTEx project (Figure 1C). The results highlight the unique expression patterns of TNNT1 in different tissues and its significant increase in multiple types of tumours.

### 3.2 Prognostic significance of TNNT1 in pan‐cancer

**FIGURE 2:**
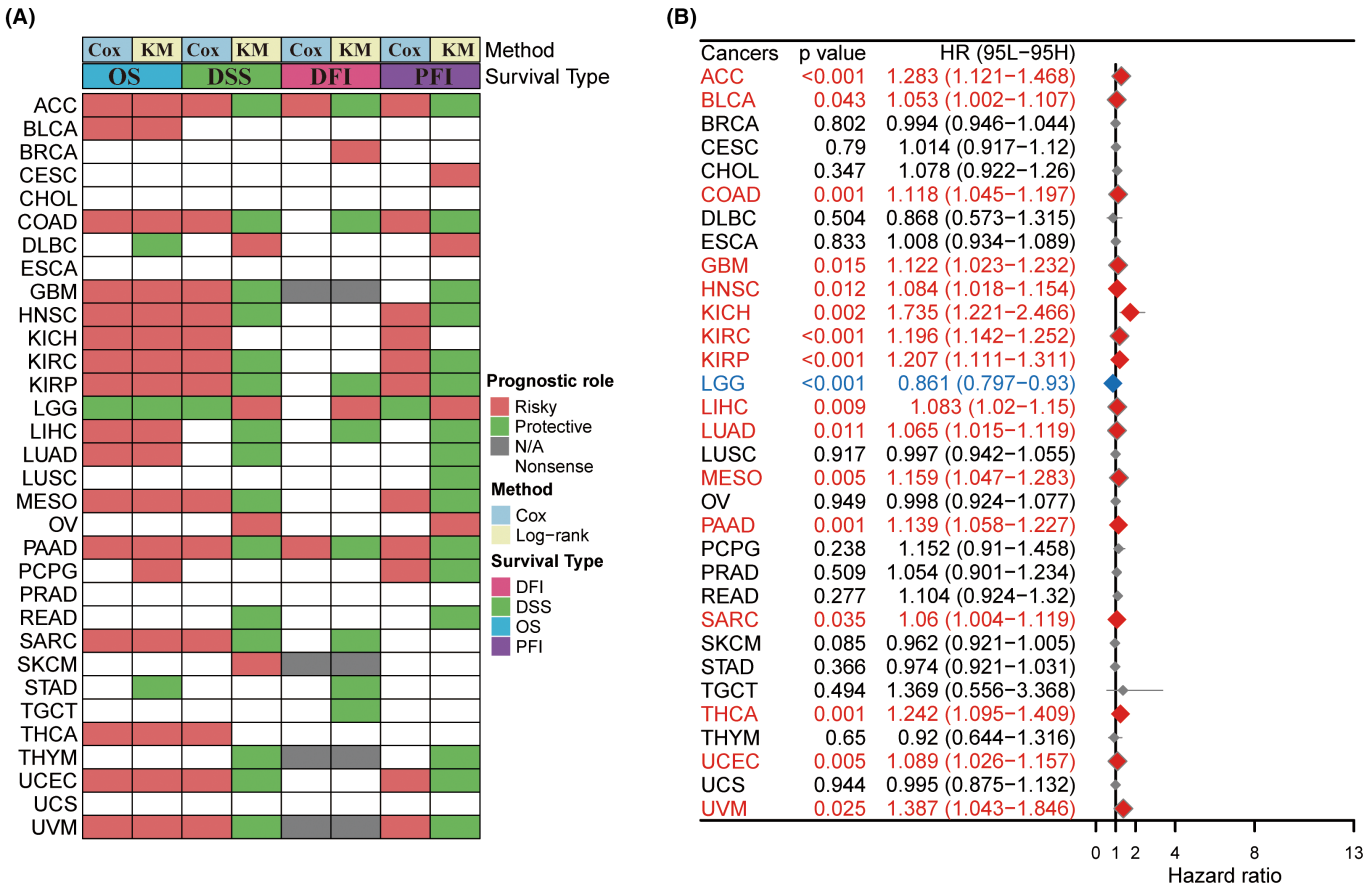
Prognostic roles of TNNT1 in pan‐cancer. (A) Summary of the correlations between CDKN2A expression and overall survival (OS), disease‐specific survival (DSS), disease‐free interval (DFI), and progression‐free interval (PFI) of cancer patients according to univariate Cox regression and Kaplan–Meier model; (B) through univariate Cox regression method, forest map shows the prognostic role of TNNT1in cancer.

We examined the TNNT1 mRNA expression levels in the UCSC database to assess their influence on cancer prognosis by investigating their associations with OS, DSS, DFI and PFI across different cancer categories. Surprisingly, TNNT1 levels were found to be a predictive factor in 25 types of tumours, with the exception of CHOL, ESCA, PRAD and UCS. Analysis of heatmap clustering revealed unique TNNT1 expression patterns associated with patient outcomes in these types of tumours (Figure 2A). Notably, elevated TNNT1 expression levels were associated with reduced OS in various types of cancer including ACC, BLCA, COAD, GBM, HNSC, KICH, KIRC, KIRP, LIHC, LUAD, MESO, PAAD, SARC, THCA, UCEC and UVM among those examined (Figure 2B). These results suggest that TNNT1 expression may serve as a promising prognostic marker in these types of cancer.

### 3.3 Genetic mutation analysis of TNNT1 across a spectrum of cancers

**FIGURE 3:**
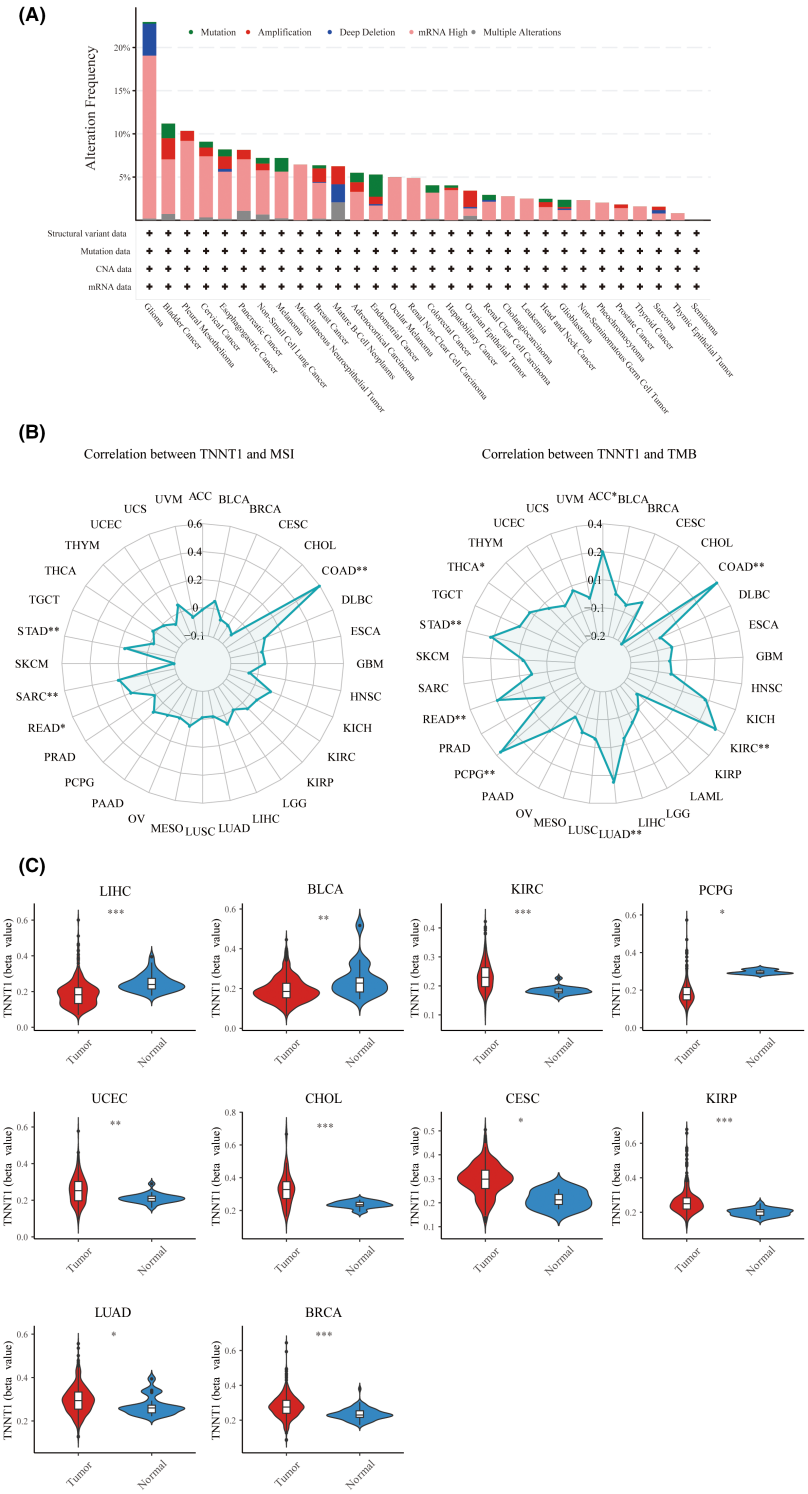
Gene alteration and methylation analysis for TNNT1 in pan‐cancer. (A) The gene alteration of TNNT1 in pan‐cancer; (B) correlation between MSI, TMB and TNNT1; (C) differential methylation analysis of TNNT1 in tumour samples versus normal ones. *p < 0.05; **p < 0.01; ***p < 0.001.

The occurrence and categorization of TNNT1 mutations were scrutinized across diverse tumour types, utilizing data sourced from the cBioPortal database (Figure 3A). Notably, gliomas manifested the highest frequency of TNNT1 mutations among the scrutinized tumours. The prevailing mutation type observed across multiple tumour types was ‘mRNA high’, signifying pervasive modifications in TNNT1 mRNA expression levels. The recognition of widespread alterations in TNNT1 mRNA expression levels underscores the potential significance of TNNT1 in the intricate process of tumorigenesis.

### 3.4 Correlation between TNNT1 expression and MSI and TMB

MSI and TMB stand as two biomarkers intricately intertwined with tumoral genomic attributes. They hold considerable significance in the realm of oncologic research and treatment. We examined how TNNT1 expression correlates with MSI or TMB levels in various tumours. Figure 3B shows that TNNT1 expression is positively correlated with MSI in COAD, STAD, SARC and READ tumours. Likewise, a positive relationship was observed between TNNT1 levels and TMB in colorectal, kidney, lung, adrenal, rectal, stomach, thyroid and adrenal cancers. These findings underscore the potential utility of TNNT1 as a biomarker indicative of the underlying genomic landscape of tumours.

### 3.5 Methylation analysis of TNNT1 in pan‐cancer

Epigenetic methylation plays a crucial part in the transformation and progression of cancer. Employing the bioinformatics tool ‘UCSCXenaShiny’, we scrutinized TNNT1 methylation levels across diverse tumour types. As delineated in Figure 3C, LIHC, BLCA and PCPG malignancies showcased conspicuously heightened TNNT1 methylation in normal tissues when juxtaposed with their corresponding tumour counterparts. Conversely, neoplastic specimens from KIRC, UCEC, CHOL, CESC, KIRP, LUAD and BRCA cancers exhibited significantly augmented TNNT1 methylation compared to normal tissues. These findings unveil distinctive TNNT1 methylation profiles between states of health and malignancy across a spectrum of cancer histologies.

### 3.6 Functional enrichment analysis of TNNT1

**FIGURE 4:**
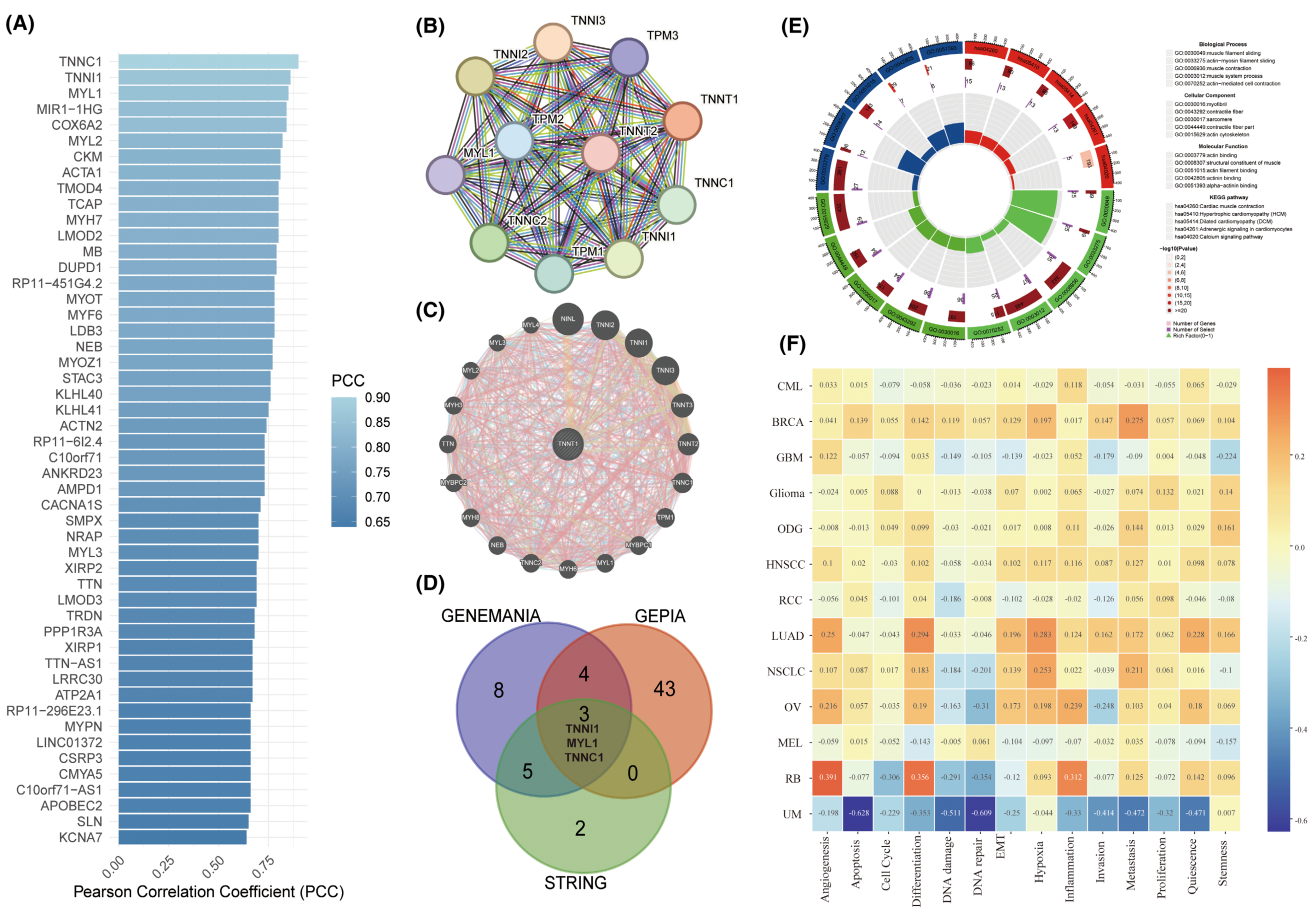
Functional enrichment analysis of TNNT1 and its functional partners. (A) Top 50 similar genes of TNNT1 (GEPIA database); (B) construction of the protein–protein interaction (PPI) network (STRING database); (C) gene network analysis (GENEMANIA database); (D) the intersection of genes obtained from the aforementioned three databases; (E) GO and KEGG analysis of all genes obtained from the aforementioned three databases; (F) the associations between cancer hallmarks and TNNT1 expression (CancerSEA database). GO, gene ontology; KEGG, Kyoto Encyclopedia of Genes and Genomes.

To further explicate the biological significance and prognostic implications of TNNT1 in cancer, we executed functional enrichment analyses employing various computational methodologies. Initially, we scrutinized TNNT1 through GEPIA2, identifying its top 50 co‐expressed genes (Figure 4A). Subsequently, we meticulously constructed comprehensive protein and gene interaction networks utilizing STRING and GENEMANIA, elucidating the intricate regulatory interactions of TNNT1 (Figure 4B,C). Remarkably, TNNI1, MYL1 and TNNC1 consistently manifested as closely interconnected with TNNT1 across our analyses (Figure 4D). Additionally, we performed GO and KEGG pathway analyses on the functional gene sets (Figure 4E). This unveiled biological processes revolving around muscle contraction and cytoarchitecture, notable actin interactions, and cellular components emphasizing myogenic fibre structural integrity. Key pathways implicated encompass cardiac muscle contraction, cardiomyopathies, adrenergic signalling in cardiomyocytes, and calcium signalling. Furthermore, scrutinizing TNNT1's function in cancer through CancerSEA, we discerned positive correlations with angiogenesis, EMT, and metastasis in HNSCC, LUAD, NSCLC, OV and RB (Figure 4F). This suggests that TNNT1 may potentiate malignancy in these tumours by fostering angiogenic, EMT, and metastatic phenotypes. In summary, our multifaceted computational analyses yield novel insights into both the physiological and cancer‐related roles of TNNT1, elucidating its regulatory interactome and involvement in critical oncogenic pathways.

### 3.7 Investigation of immune cell infiltration in TNNT1 across various types of cancer

**FIGURE 5:**
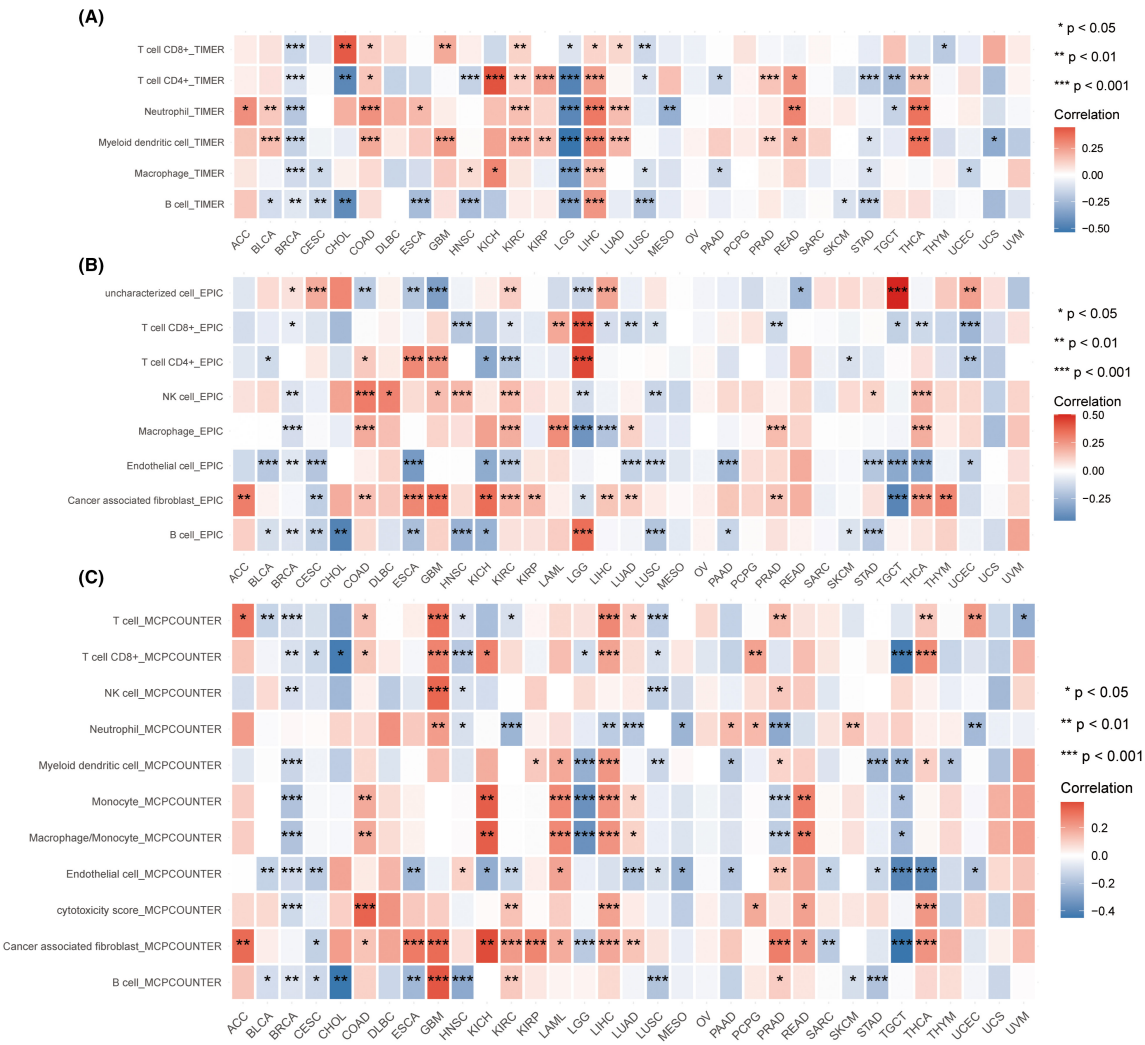
Pan‐cancer analysis of the correlation between TNNT1 expression and immune cell infiltration using different algorithms. (A) TIMER algorithm; (B) EPIC algorithm; (C) MCPCOUNTER algorithm. *p < 0.05; **p < 0.01; ***p < 0.001.

Derived from analyses in the TIMER, EPIC and MCPcounter databases, we have systematically elucidated the immune regulatory and TME modulation functions associated with TNNT1 across diverse cancer types. The TIMER analysis showed a strong connection between TNNT1 levels and the presence of dendritic cells and neutrophils in various cancer types, such as BLCA, COAD, KIRC, LIHC, LUAD, READ and THCA (Figure 5A). EPIC and MCPcounter analyses consistently showed a strong connection between TNNT1 expression and cancer‐associated fibroblasts in various types of cancer including ACC, COAD, GBM, ESCA, KICH, KIRC, KIRP, LIHC, LUAD, PRAD and THCA, as illustrated in Figure 5B,C. Collectively, these findings suggest that TNNT1 intricately modulates the TME by influencing the infiltration dynamics of immune cells, including dendritic cells, neutrophils, and cancer‐associated fibroblasts.

### 3.8 Drug sensitivity analysis of TNNT1

**FIGURE 6:**
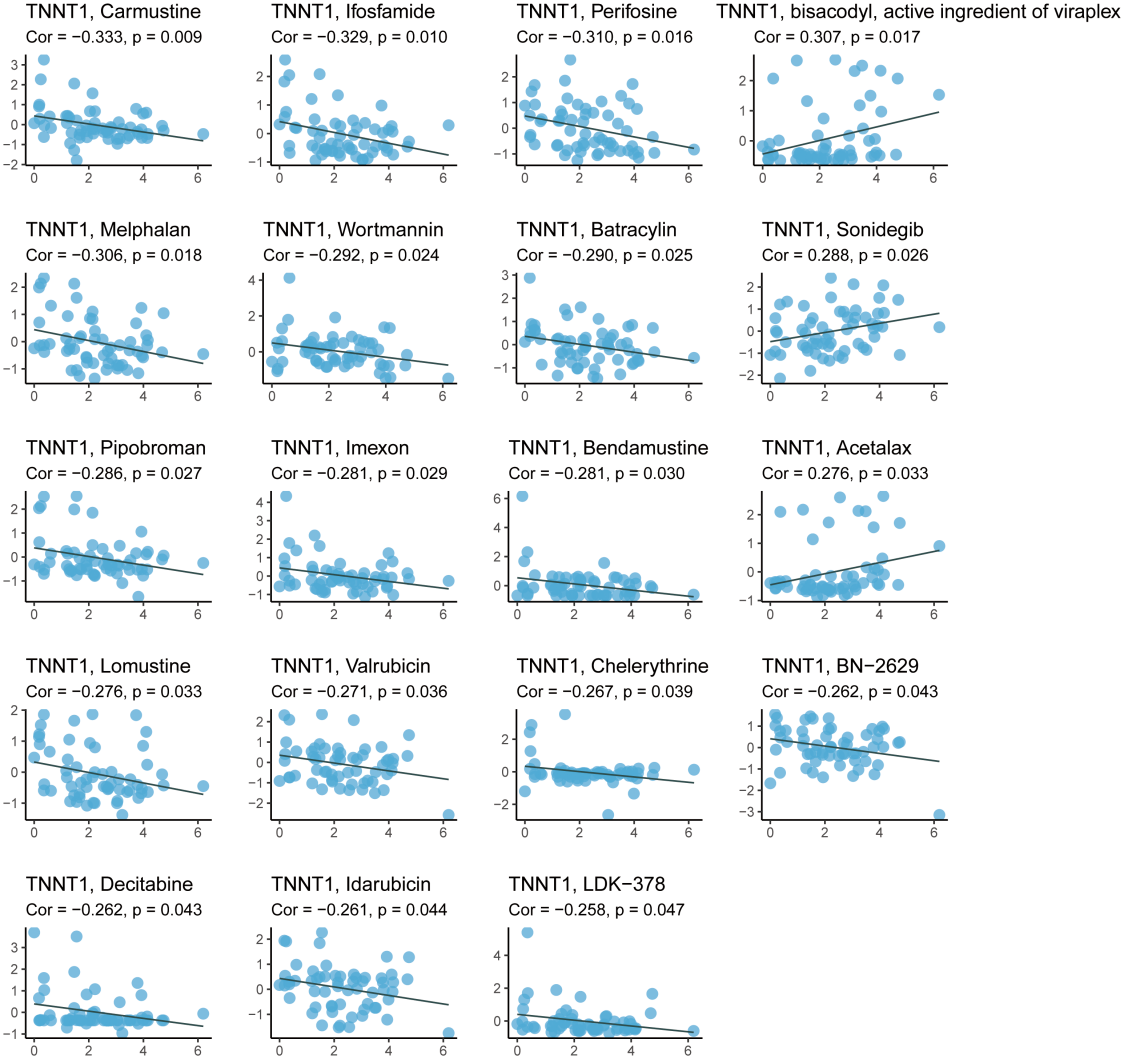
TNNT1 expression was shown to be closely associated with IC50 values of 19 drugs (CellMiner database).

We examined drug sensitivity data extracted from the CellMiner database and identified significant associations between TNNT1 expression levels and the IC50 values of specific chemotherapeutic agents (Figure 6). In the case of chemotherapeutics such as carmustine, ifosfamide, melphalan, batracylin, pipobroman, bendamustine, lomustine, valrubicin and decitabine, heightened TNNT1 levels correlated with diminished IC50 (indicating increased sensitivity). Conversely, for sonidegib, elevated TNNT1 levels were linked to elevated IC50 (indicating reduced sensitivity). These findings indicate that TNNT1 may have a two‐fold effect on how tumour cells react to chemotherapy. It appears to augment chemosensitivity while concurrently diminishing chemotherapy sensitivity, particularly in the context of sonidegib.

### 3.9 High levels of TNNT1 expression are associated with more advanced clinicopathological characteristics in patients with KIRC

**FIGURE 7:**
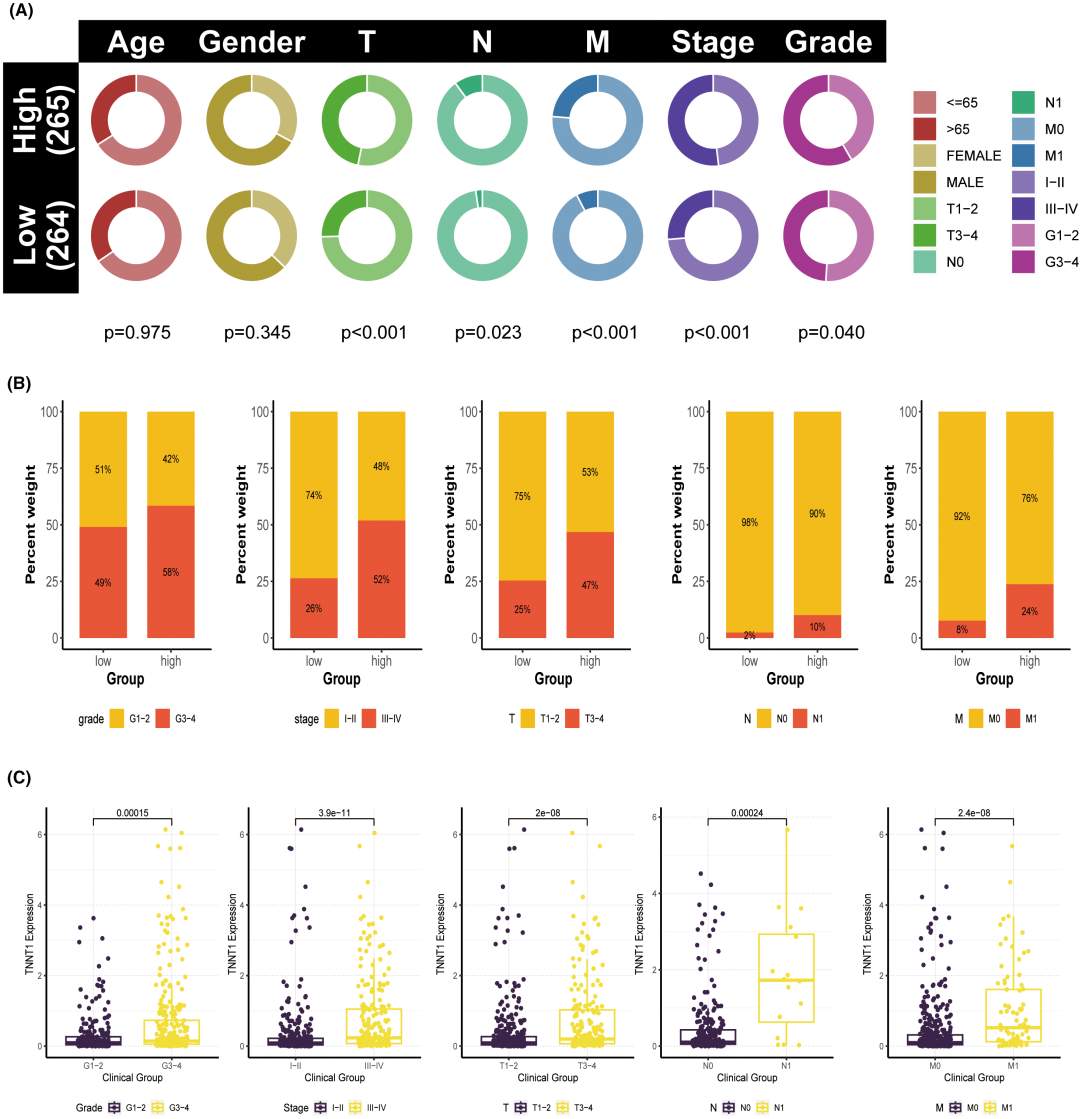
Clinical correlation analysis of TNNT1 expression in kidney renal clear cell carcinoma (KIRC). (A, B) The high TNNT1 group exhibited associations with higher grade (G3–G4), advanced stage (III–IV), larger tumour size (T3–T4), lymph node metastasis (N1), and distant metastasis (M1); (C) high expression of TNNT1 was observed in patients with advanced disease characteristics, including G3–G4 grade, stage III–IV, T3–T4 tumour size, N1 lymph node metastasis, and M1 distant metastasis.

Due to the increased TNNT1 expression and its link to a poor prognosis in KIRC, we further investigated the relationship between TNNT1 levels and clinicopathological features in KIRC patients using information from the TCGA group. Results showed a higher occurrence of severe disease characteristics, such as higher grade (G3–G4), advanced stage (III–IV), larger tumour size (T3–T4), lymph node involvement (N1), and distant metastasis (M1), in patients with increased TNNT1 levels compared to those with lower TNNT1 levels (Figure 7A,B). Individuals with more severe disease characteristics (G3–G4 grade, stage III–IV, T3–T4, N1, M1) showed higher levels of TNNT1 expression compared to those with less severe features (G1–G2 grade, stage I–II, T1–T2, N0, M0), as shown in Figure 7C. The results suggest that TNNT1 expression could serve as a biomarker to assess the advancement and severity of disease in KIRC.

### 3.10 Prognosis role and clinical value of TNNT1 in KIRC

**FIGURE 8:**
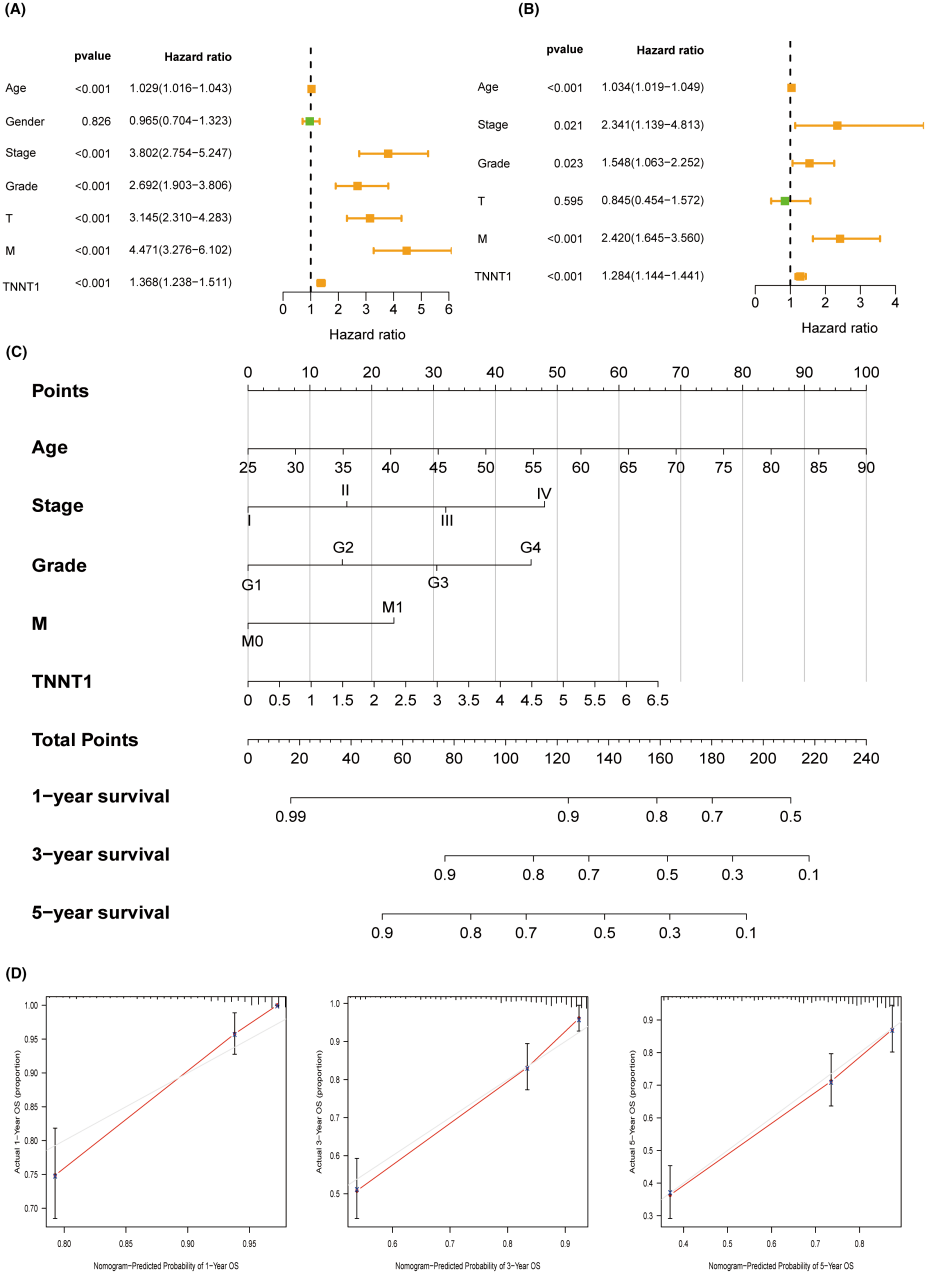
Clinical value evaluation of TNNT1 in kidney renal clear cell carcinoma (KIRC). (A, B) The results of univariate independent prognostic analysis and multivariate independent prognostic analysis; (C, D) developed nomogram and calibration curves of the nomogram.

Univariate and multivariate Cox regression were performed to evaluate the significance of TNNT1 expression as an independent risk factor in KIRC. Figure 8A,B delineate the findings that age, stage, grade, metastasis and TNNT1 expression are independent prognostic factors for KIRC. Utilizing this information, a nomogram was created that included the five identified risk factors from the multivariate analysis to forecast the 1, 3, and 5‐year OS rates of patients with KIRC (Figure 8C). The calibration curve of the nomogram exhibited a notably high level of concordance between the anticipated and actual probabilities of survival (Figure 8D). These findings establish TNNT1 as an innovative and independent prognostic marker for KIRC.

### 3.11 Functional enrichment analysis of TNNT1 in KIRC

**FIGURE 9:**
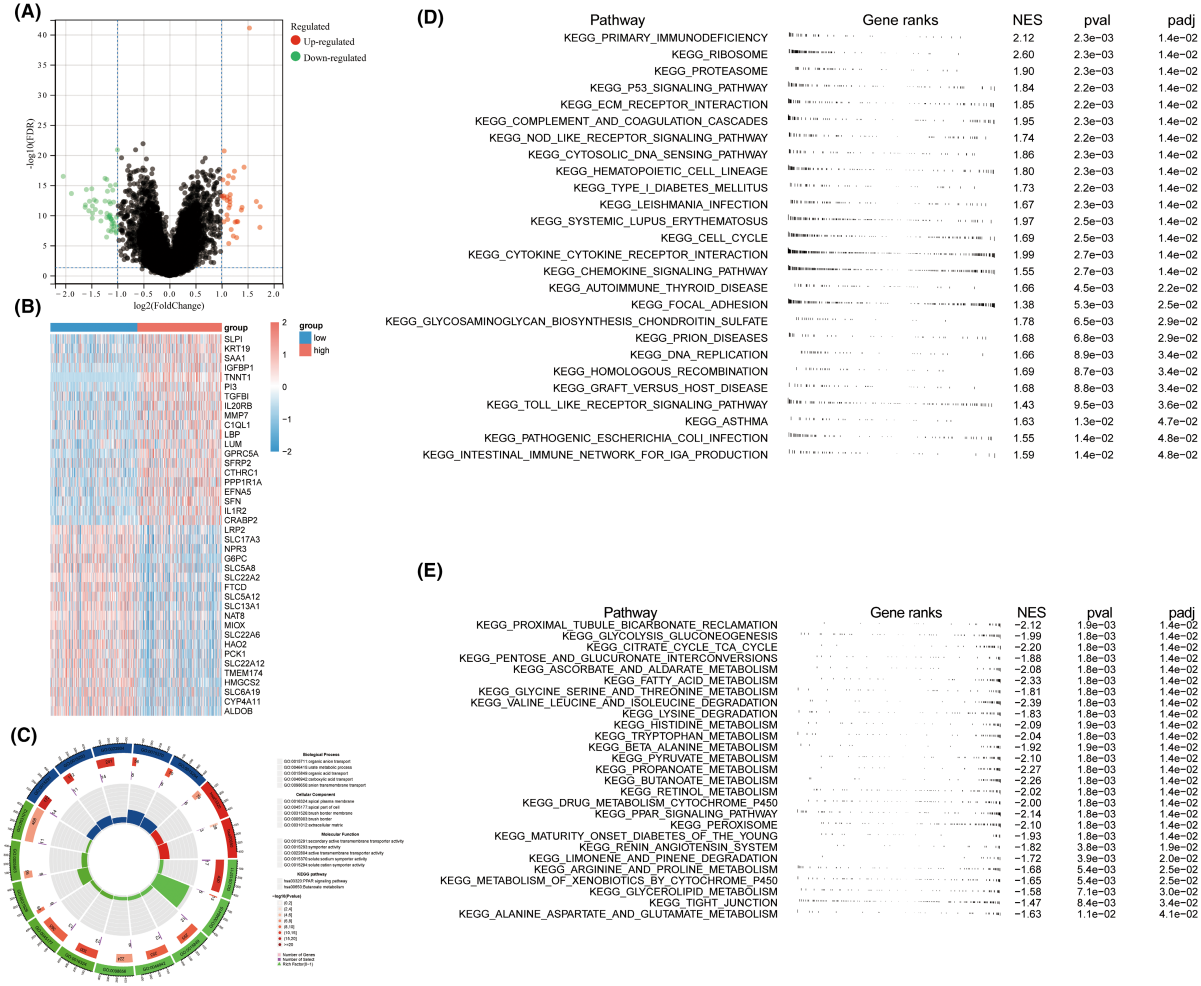
Potential functions of TNNT1 in kidney renal clear cell carcinoma (KIRC). (A) Volcano plot of differentially expressed genes (DEGS) between high and low TNNT1 expression groups; (B) heatmap of top 20 DEGs between high and low TNNT1 expression groups; (C) go and KEGG analysis of DEGs between high and low TNNT1 expression groups; (D) The fgsea results of activated Kyoto Encyclopedia of Genes and Genomes (KEGG) pathways in KIRC; (E) the fgsea results of inhibited KEGG pathways in KIRC. NES, normalized enrichment score; pval, *p* value; padj, adjusted *p* value.

KIRC patients were categorized into two groups based on the median TNNT1 expression value, with one group showing high expression and the other group showing low expression. The categorization identified 83 Genes with Differential Expression (DEGs) between the two groups (|log (Fold Change)|>1 and False Discovery Rate <0.05) (Figure 9A). Heatmaps were constructed for the top 20 DEGs, depicted in Figure 9B. Enrichment analysis results are elucidated in Figure 9C. Notably, the DEGs demonstrated significant enrichment in biological processes, encompassing organic anion transport and urate metabolic processes. Moreover, they exhibited enrichment in cellular components such as the apical plasma membrane and brush border, along with molecular functions like secondary active transmembrane transport activity. Additionally, these genes displayed enrichment in KEGG pathways, particularly in PPAR signalling and butyrate metabolism. The GSEA findings showed that the high TNNT21 expression group had enrichment in pathways related to cancer and the immune system (Figure 9D). In contrast, samples exhibiting low TNNT1 expression showed a significant enrichment of pathways associated with important metabolic functions like glycolysis, TCA cycle, fatty acid metabolism, and amino acid degradation (Figure 9E). The results suggest a connection between the TNNT1 expression level in KIRC patients and various molecular and functional traits, such as differences in cancer‐related pathways, immune‐related pathways, and metabolic processes.

### 3.12 Tumour microenvironment characterization of TNNT1 in KIRC

**FIGURE 10:**
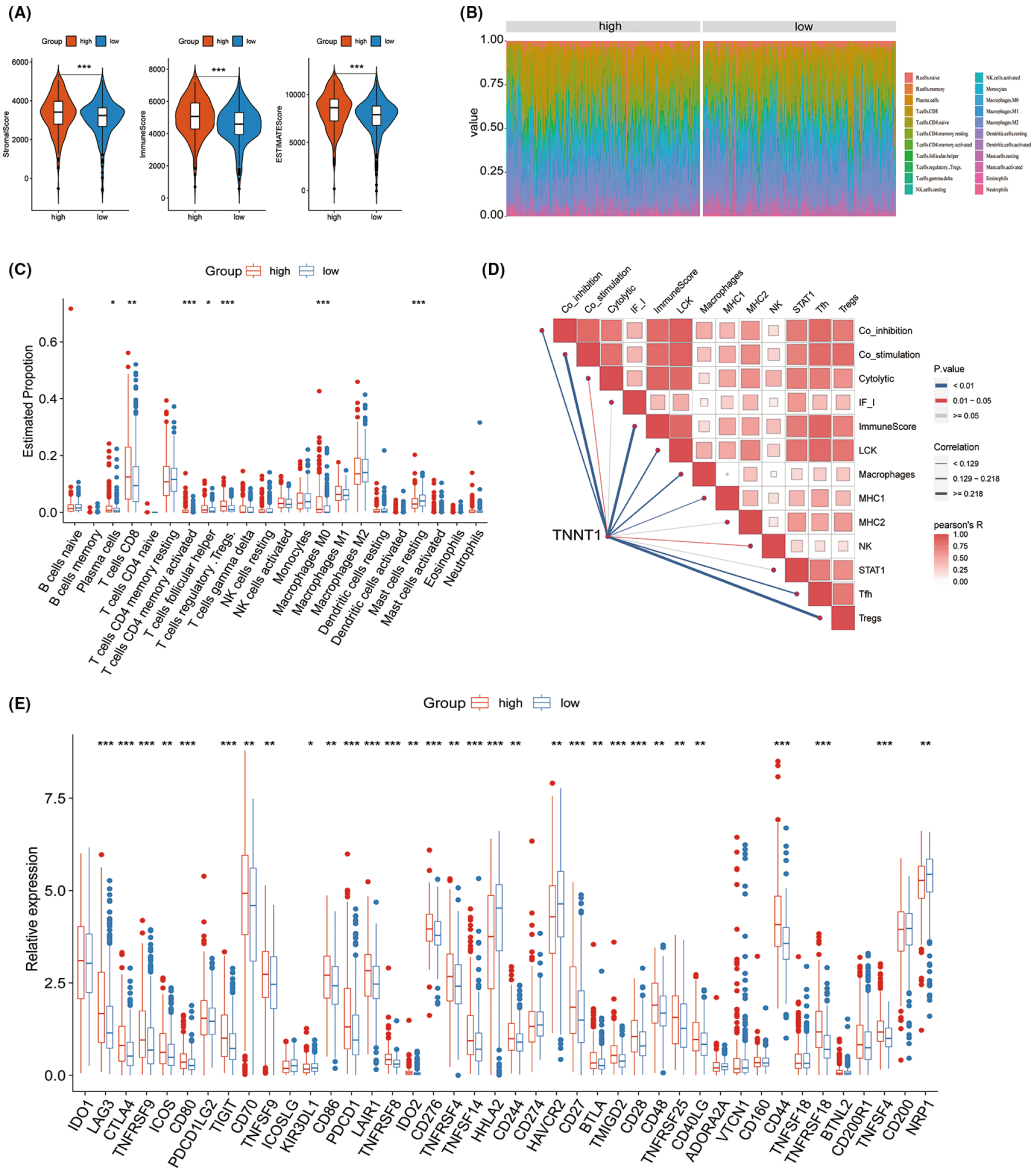
The immune infiltration landscape of TNNT1 in kidney renal clear cell carcinoma (KIRC). (A) Differences in tumour microenvironment (TME) scores between TNNT1 high‐ and low‐expression groups; (B) the proportions of 22 infiltrating immune cell types in KIRC samples; (C) the difference in immune cell infiltration between low‐ and high‐expression TNNT1 groups; (D) association of TNNT1expression with the infiltration of immune cells and functions (ssGSEA); (E) differences in the gene expression of immune checkpoint related genes between high and low TNNT1 expression groups. *p < 0.05; **p < 0.01; ***p < 0.001.

According to the results of the GSEA analysis, the group of KIRC patients with high levels of TNNT1 showed enrichment in pathways related to chemokine signalling and processes associated with adaptive immunity. This implies that TNNT1 may exert influence in orchestrating the TME within KIRC. To delve deeper into the role of TNNT1 in the KIRC TME, multiple analyses were conducted. Initially, TME scores were assessed using the ESTIMATE algorithm in the TCGA‐KIRC cohort. The results showed that samples with high TNNT1 expression had higher stromal scores, immune scores, and overall ESTIMATE scores in comparison to samples with low TNNT1 expression (Figure 10A). Subsequently, the quantification of immune cell components within the TME was executed using the CIBERSORT method Figure 10B. shows a histogram that displays the distribution of 22 different types of infiltrating immune cells in every sample. Variances in immune cell penetration were observed in the groups with high and low levels of TNNT1 expression. In particular, specimens from the group with elevated TNNT1 levels exhibited increased percentages of Plasma cells, CD8 T cells, activated memory CD4 T cells, follicular helper T cells, Tregs and M0 Macrophages. In contrast, the proportion of inactive Mast cells was reduced in the group with elevated TNNT1 levels (Figure 10C). The ssGSEA findings showed a significant increase in the presence of Tfh, Tregs, NK cells, MHC1, macrophages, LCK, ImmuneScore, Cytolytic, Co‐inhibition and Co‐stimulation as the expression of TNNT1 rose (Figure 10D). Upon examining ICI‐associated genes in high and low expression groups, it was found that most ICI‐related genes showed increased expression in the high TNNT1 cohort. Specifically, in the high TNNT1 group, genes like CTLA4, PDCD1 (which encodes PD‐1), and LAG3 showed elevated levels of expression (see Figure 10E). The findings suggest that TNNT1 could play a crucial part in regulating the TME, promoting the infiltration of immune cells, and impacting the expression of immune checkpoint proteins in KIRC.

### 3.13 Drug sensitivity and immunotherapy

**FIGURE 11:**
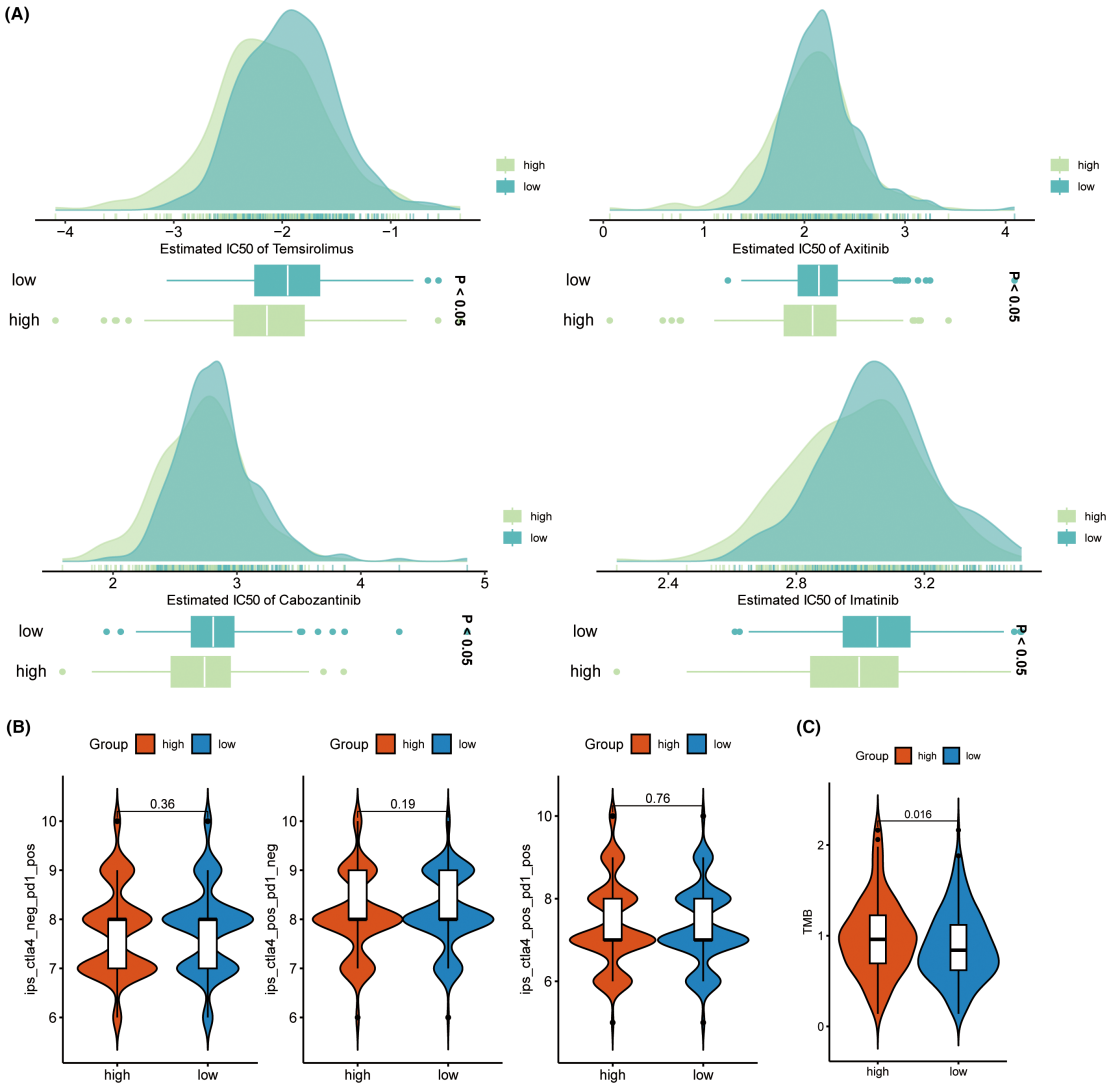
Drug sensitivity and immunotherapy of TNNT1 in kidney renal clear cell carcinoma (KIRC). (A) IC50 values of temsirolimus, axitinib, cabozantinib and imatinib were lower in TNNT1 high expression group compared to TNNT1 low expression group; (B) immunophenoscore (IPS) for immunotherapy; (C) TMB difference in the low‐ and high‐expression TNNT1 groups.

Pharmaceutical analysis was conducted on KIRC samples with different TNNT1 expression levels to examine the influence of TNNT1 expression on drug response in KIRC. Results showed that the group with increased TNNT1 levels had significantly lower IC50 values for temsirolimus, axitinib, cabozantinib and imatinib, indicating increased sensitivity to these drugs compared to the group with lower TNNT1 levels (Figure 11A). Recent studies propose that IPS based on immunogenicity holds utility in predicting responses to immunotherapy. We assessed immunotherapy response through IPS (Figure 11B). Nevertheless, we observed no noticeable variation in results between individuals receiving ICI treatment (CTLA4+/PD‐1‐, CTLA4‐/PD‐1+, and CTLA4+/PD‐1+). TMB is used to measure the number of genetic mutations in a tumour's DNA, and it is recognized as a reliable indicator of how well immunotherapy will work. Typically, heightened TMB correlates with enhanced responsiveness to immunotherapy. During our examination, we studied the relationship between TMB and TNNT1 levels in KIRC, finding that individuals with higher TNNT1 expression had higher TMB levels than those with lower TNNT1 expression (see Figure 11C). This implies that people with increased TNNT1 levels might have a better response to immunotherapy.

### 3.14 Single‐cell analysis of TNNT1 in KIRC

**FIGURE 12:**
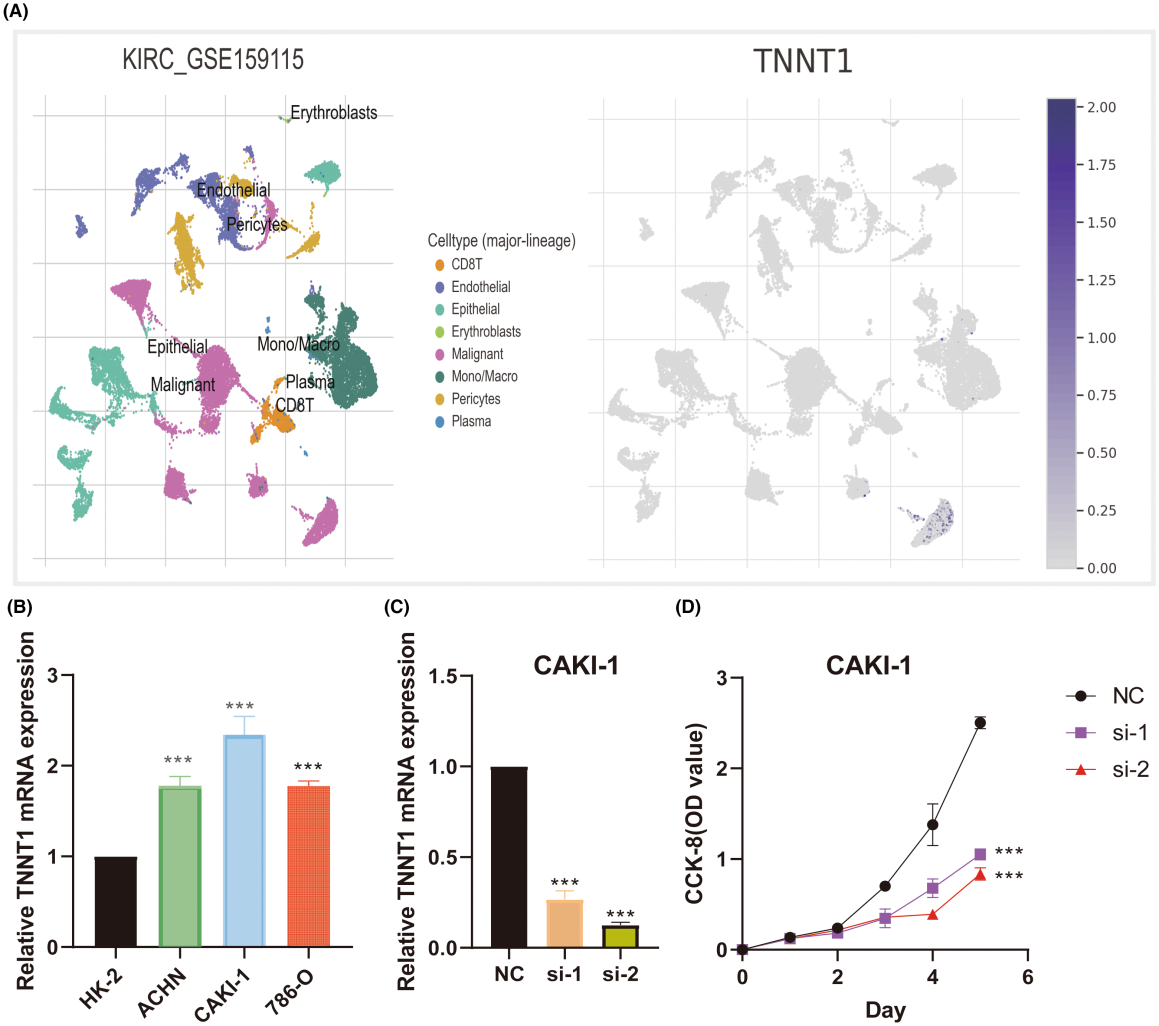
Single‐cell analysis and CCK‐8 proliferation experiments. (A) Cell localization of TNNT1 in single‐cell dataset of KIRC (GSE159115) (TISCH database); (B) comparison of TNNT1 expression level in cancer cell line and normal cell line; (C) quantitative real‐time polymerase chain reaction (qRT‐PCR) confirmation of TNNT1 knockdown in CAKI‐1 cells; (D) CCK8 experiments demonstrate the effect of TNNT1 knockdown on the proliferation of CAKI‐1 cells. ***p < 0.001.

To ascertain TNNT1 expression patterns in KIRC, we meticulously investigated the distinct expression of TNNT1 within immune and malignant cellular populations, employing the TISCH tool. As delineated in Figure 12A, TNNT1 exhibited robust upregulation in malignant cells. This observation signifies a conspicuous elevation in TNNT1 expression specifically within the malignant cellular component of KIRC.

### 3.15 TNNT1 knockdown inhibits cancer cell proliferation

We used qRT‐PCR to assess the natural expression of TNNT1 in KIRC cell lines. We noticed a significantly increased TNNT1 expression in KIRC cells compared to HK‐2 cells (Figure 12B). Due to the highest level of TNNT1 expression in CAKI‐1 cells, we chose this specific cell line for further studies. In order to clarify the importance of TNNT1, we conducted a specific reduction of TNNT1 in CAKI‐1 cells (Figure 12C) and carried out cell viability tests using the CCK‐8 assay to evaluate the growth potential of TNNT1‐depleted CAKI‐1 cells compared to the control group. Surprisingly, our results showed a significant decrease in the ability of CAKI‐1 cells to multiply after TNNT1 knockdown (Figure 12D).

## 4 DISCUSSION

TNNT1, also referred to as cardiac troponin T, is essential in regulating the interaction between actin and myosin filaments during striated muscle contraction. Previous studies have indicated its critical function in cardiomyocyte physiology and cardiac contractility. However, the participation of TNNT1 in tumorigenesis remains largely elusive.

In this study, we have thoroughly investigated the expression profiles, medical relevance, biological roles and control mechanisms of TNNT1 across various cancers using a range of computational methods. Distinct tissue‐specific and cancer‐associated expression profiles of TNNT1 were identified through comprehensive bioinformatics analyses. Significantly, conspicuous upregulation of TNNT1 was observed across multiple tumour types in comparison to normal tissues. Subsequent survival analyses revealed that elevated TNNT1 mRNA levels correlated with diminished OS in 25 cancer types. Specifically, elevated levels of TNNT1 indicated a poor outlook in ACC, BLCA, COAD, GBM, HNSC, KICH, KIRC, KIRP, LIHC, LUAD, MESO, PAAD, SARC, THCA, UCEC and UVM. These findings establish the clinical significance of TNNT1 as a prognostic biomarker in diverse neoplasms.

Furthermore, we discerned frequent modifications in TNNT1 expression levels across cancer types through mutation analyses utilizing cBioPortal. Aberrant methylation patterns were also uncovered through meticulous epigenomic profiling across tumours and paired normal tissues. Differential TNNT1 methylation distinguished healthy tissues from malignant specimens in various organs like LIHC, BLCA, PCPG, KIRC, UCEC, CHOL, CESC, KIRP and LUAD. Dysregulated methylation holds the potential to drive the aberrant TNNT1 expression witnessed in multiple cancers.

To understand the functional implications of TNNT1, we performed comprehensive bioinformatics analyses. GEPIA2 profiling identified TNNI1, MYL1 and TNNC1 as TNNT1's most co‐expressed genes. Integrative interactome modelling using STRING and GENEMANIA portrayed intricate regulatory interactions centered on TNNT1. Enrichment analyses based on these gene networks unveiled biological processes associated with muscle contraction and cytoarchitecture. The identification of key pathways implicated in our analysis centered around cardiac muscle physiology and cardiomyopathies. These findings provide insights into the potential involvement of TNNT1 in cardiac‐related processes. Moreover, through CancerSEA profiling, we observed positive associations between TNNT1 expression and angiogenic, EMT, and metastatic phenotypes specifically in HNSCC, LUAD, NSCLC, OV and RB. These findings suggest that TNNT1 may have oncogenic functions beyond its native role in striated muscle, contributing to cancer hallmarks and disease progression.

Analysis of immune cell infiltration patterns revealed a strong association between TNNT1 levels and the presence of dendritic cells and neutrophils in various types of cancer according to TIMER. These immune cells play crucial roles in the TME, with dendritic cells contributing to antigen presentation and activation of immune responses, and neutrophils involved in inflammation and immune regulation. Moreover, our findings demonstrated a favourable relationship between TNNT1 levels and the proportions of cancer‐associated fibroblasts, as supported by concordant results obtained using EPIC and MCPcounter algorithms. Cancer‐associated fibroblasts are known to play diverse roles in tumour growth, angiogenesis and immune modulation. By elucidating the association between TNNT1 expression and the presence of these immune cells and cancer‐associated fibroblasts, our study provides insights into the potential role of TNNT1 in modulating immune responses within tumours. These findings suggest that TNNT1 may contribute to the complex interplay between tumour cells, immune cells, and the TME. Drug sensitivity profiling employing the CellMiner platform revealed disparate correlations between TNNT1 levels and IC50 values of specific chemotherapeutic agents. Interestingly, higher TNNT1 levels were associated with augmented chemosensitivity to multiple drugs, indicating a potential role for TNNT1 in enhancing tumour susceptibility to these agents. However, we observed a paradoxical finding where higher TNNT1 levels were correlated with diminished responsiveness to sonidegib therapy. This suggests a complex, context‐dependent role for TNNT1 in modulating tumour responses to anti‐cancer agents. To provide further insights into the mechanisms underlying these associations, we speculate that TNNT1 may influence signalling pathways involved in drug sensitivity or resistance. For instance, TNNT1 might interact with key molecules involved in drug transport, metabolism, or DNA repair pathways, thereby affecting tumour cell response to specific chemotherapeutic agents. Investigating these potential mechanisms would be valuable for a comprehensive understanding of TNNT1's impact on drug responsiveness.

In the analysis of the TCGA cohort, we observed strong correlations between increased TNNT1 levels and poor clinical characteristics and more advanced pathological factors in KIRC.TNNT1 emerged as an autonomous prognostic indicator for KIRC through univariate and multivariate Cox regression modelling. Additionally, the nomogram incorporating TNNT1 provided accurate risk stratification and prognosis prediction for KIRC patients. Eighty‐three differentially expressed genes associated with transport and metabolism processes were identified in KIRC cohorts based on TNNT1 levels. GSEA indicated enrichment of cancer hallmark pathways and immune‐related signatures specifically in the high TNNT1 group. Conversely, metabolic pathways featured enrichment in the low TNNT1 cohort. The results suggest that increased TNNT1 levels are associated with unique molecular features in KIRC.

Assessment of the KIRC TME utilizing ESTIMATE unveiled comparatively elevated stromal scores, immune scores, and overall TME infiltration in samples with heightened TNNT1. CIBERSORT analyses divulged an immunosuppressive landscape favouring Tregs, Tfh cells and pro‐tumoral M2 macrophages with elevated TNNT1. ssGSEA outcomes reinforced a stronger immunogenic profile linked to higher TNNT1 expression. Collectively, these findings propose that TNNT1 actively orchestrates an immunosuppressive microenvironment in KIRC. Evaluation of drug responsiveness in KIRC intimated greater sensitivity to targeted therapies like temsirolimus, axitinib and cabozantinib in the high TNNT1 cohort. Additionally, elevated TNNT1 correlated with heightened TMB, a predictive marker for immunotherapy efficacy. Based on the previously mentioned research findings, high TNNT1 expression is associated with increased TMB, which is a predictive marker for immunotherapy efficacy. This suggests that immunotherapy may be more effective in KIRC patients with high TNNT1 expression. Given that TNNT1 actively regulates the immunosuppressive TME in KIRC, further research can explore interventions targeting TNNT1 to enhance the responsiveness of immunotherapy. For example, inhibiting TNNT1 may help reduce the number or functionality of immune suppressor cells, such as Tregs and M2 macrophages, thereby restoring the immune system's ability to attack the tumour. In addition, combining targeted therapies with immunotherapy is also worth investigating. KIRC patients with high TNNT1 expression have shown increased sensitivity to targeted therapy drugs such as temsirolimus, axitinib and cabozantinib. By combining targeted therapies with immune stimulants or ICIs, it may be possible to further enhance the effectiveness of immunotherapy and improve patient survival rates and treatment responsiveness.

Single‐cell profiling utilizing TISCH revealed robust TNNT1 upregulation specifically within the malignant cellular population of KIRC. Experimentally, KIRC cell lines manifested elevated endogenous TNNT1 compared to normal renal cells. Targeted TNNT1 knockdown in CAKI‐1 cells significantly suppressed cancer cell proliferation in vitro. These findings demonstrate an oncogenic function of TNNT1 in directly promoting KIRC tumorigenesis.

The current study provides the most comprehensive investigation of TNNT1's pan‐cancer profiling, clinical impact, biological functions and underlying regulatory mechanisms to date. Several novel insights emerge—TNNT1 exhibits differential expression patterns distinguishing healthy and malignant states; elevated levels portend an unfavourable prognosis across cancers; intricate protein interaction networks implicate its role beyond muscle; immune modulation functions influence TME dynamics; variable correlations with drug responses imply context‐specific roles. Regarding KIRC, our data establishes TNNT1 as a promising prognostic biomarker, showing a strong association with advanced disease features, an immunosuppressive microenvironment, and sensitivity to targeted therapies. Specifically, our study provides experimental validation of TNNT1's oncogenic functions in directly promoting KIRC cell proliferation, further substantiating the evidence for its clinical relevance. Considering the potential clinical ramifications of our discoveries, TNNT1's expression or mutational status could serve as valuable indicators to guide therapeutic decisions and foster personalized treatment protocols in KIRC. For instance, high TNNT1 expression may indicate a poor prognosis and the need for more aggressive treatment strategies. Additionally, targeting TNNT1 or its associated signalling pathways could be explored as a potential therapeutic approach in KIRC. Furthermore, the incorporation of TNNT1 into a nomogram provides a practical clinical application, enabling risk assessment and prognostication for KIRC management. This tool could assist clinicians in making informed decisions and tailoring treatment plans based on individual patient characteristics and TNNT1 expression levels.

The major caveat of this investigation is its retrospective nature, which relies on bioinformatics analyses of existing patient cohorts. While our study provides valuable insights into the potential clinical significance and prognostic potential of TNNT1, it is important to acknowledge the limitations in establishing causality or directly informing clinical decision making. To further elucidate TNNT1's role in oncology and address these limitations, future research should incorporate prospectively designed experimental and interventional studies. These studies would enable a more comprehensive evaluation of TNNT1's functional mechanisms, causality relationships, and potential therapeutic implications. Additionally, investigating TNNT1 in larger patient cohorts with longitudinal follow‐up would provide robust evidence regarding its clinical significance and prognostic value.

In summary, through multidimensional bioinformatics profiling and preliminary experimental verifications, our study establishes TNNT1 as a prognostic biomarker for diverse cancers, especially KIRC. Novel insights into its biological roles beyond muscle physiology, immunomodulatory functions and ability to influence tumour drug responses provide valuable avenues for future mechanistic investigations. Altogether, our findings substantiate the clinical relevance and functional significance of TNNT1 in cancer, proposing it as a promising candidate for precision oncology applications.

## AUTHOR CONTRIBUTIONS

**Qianjin Zhang:** Conceptualization (equal); writing – original draft (equal). **Lin Hao:** Conceptualization (equal); writing – original draft (equal). **Fengye Wang:** Conceptualization (equal); investigation (equal); visualization (equal). **Quansheng Yu:** Formal analysis (equal); validation (equal). **Shaoyuan Wu:** Formal analysis (equal); software (equal). **Conghui Han:** Project administration (equal); supervision (equal); writing – review and editing (equal).

## FUNDING INFORMATION

This study was funded by Suqian Sci & Tech Program, grant no. KY202205, and Suqian First Peoples Hospital 136 Youth Talent Program, grant no. 38.

## CONFLICT OF INTEREST STATEMENT

The authors confirm that there are no conflicts of interest.

## Supporting information


**Figure S1:** Flow chart of this study.

Table S1:

Table S2:

## Data Availability

The data that support the findings of this study are available on request from the corresponding author.
