# Supplementary figures and images for: Troponin T1 in tumorigenesis and immune modulation: Insights into multiple cancers and kidney renal clear cell carcinoma
Source: J Cell Mol Med. 2024 Jun 9;28(11):e18410. doi: 10.1111/jcmm.18410 (PMC11163025; doi:10.1111/jcmm.18410)

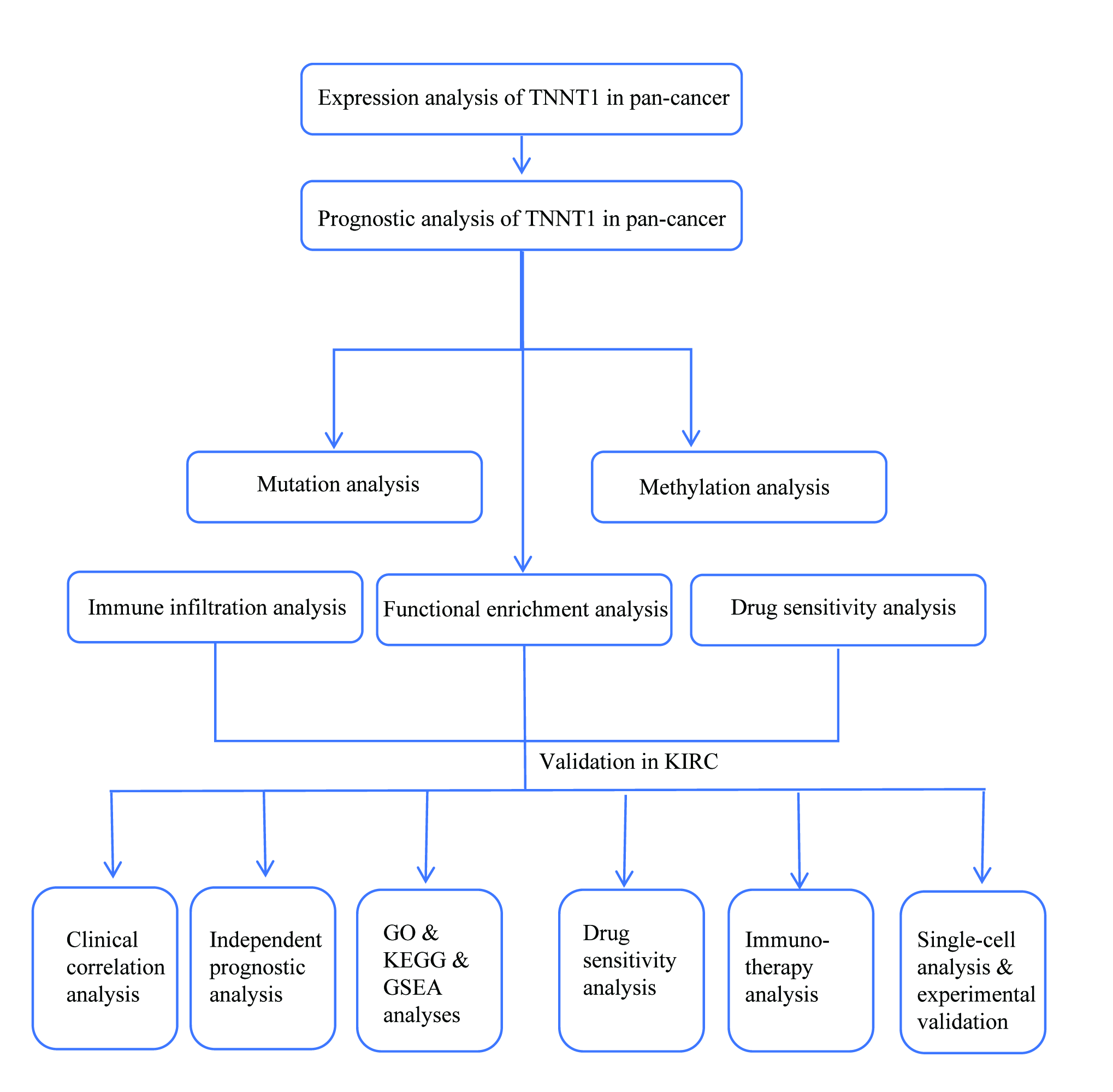

Supplement: Supplementary file 1 — Figure S1: Flow chart of this study. [file JCMM-28-e18410-s001.tif]
