# Supplementary material for: Troponin T1 in tumorigenesis and immune modulation: Insights into multiple cancers and kidney renal clear cell carcinoma
Source: J Cell Mol Med. 2024 Jun 9;28(11):e18410. doi: 10.1111/jcmm.18410 (PMC11163025; doi:10.1111/jcmm.18410)
Supplement: Supplementary file 2 — Table S1: [file JCMM-28-e18410-s002.docx]

**Table S1. The primer sequences for qRT-PCR.**

| **Gene** | **Sequences (5’>3’)** |
| --- | --- |
| TNNT1-F | TGATCCCGCCAAAGATCCC |
| TNNT1-R | TCTTCCGCTGCTCGAAATGTA |
| GAPDH-F | GAAGGTGAAGGTCGGAGT |
| GAPDH-R | GAAGATGGTGATGGGATTTC |
