# Supplementary material for: Troponin T1 in tumorigenesis and immune modulation: Insights into multiple cancers and kidney renal clear cell carcinoma
Source: J Cell Mol Med. 2024 Jun 9;28(11):e18410. doi: 10.1111/jcmm.18410 (PMC11163025; doi:10.1111/jcmm.18410)
Supplement: Supplementary file 3 — Table S2: [file JCMM-28-e18410-s003.docx]

**Table S2. The siRNA sequences used in this study.**

| **Gene** | **Sequences (5’>3’)** |
| --- | --- |
| siTNNT1#1 | GCTGAAACAGCAGAAATATGA |
| siTNNT1#2 | GTCACCAATAATCATTAAAGTAC |
